# Shape Programming Using Triangular and Rectangular Soft Robot Primitives

**DOI:** 10.3390/mi10040236

**Published:** 2019-04-07

**Authors:** Phone May Khin, Jin Huat Low, Marcelo H. Ang, Chen-Hua Yeow

**Affiliations:** 1 Department of Biomedical Engineering, National University of Singapore, Singapore 117583, Singapore; biekpm@nus.edu.sg; 2 Singapore Institute for Neurotechnology, National University of Singapore, Singapore 117456, Singapore; lsiljh@nus.edu.sg; 3 Department of Mechanical Engineering, National University of Singapore, Singapore 117575, Singapore; mpeangh@nus.edu.sg

**Keywords:** flexible structures, soft pneumatic actuators, fabric-based soft actuator

## Abstract

This paper presents fabric-based soft robotic modules with primitive morphologies, which are analogous to basic geometrical polygons—trilateral and quadrilateral. The two modules are the inflatable beam (IB) and fabric-based rotary actuator (FRA). The FRA module is designed with origami-inspired V-shaped pleats, which creates a trilateral outline. Upon pressurization, the pleats unfold, which enables propagation of angular displacement of the FRA module. This allows the FRA module to be implemented as a mobility unit in the larger assembly of pneumatic structures. In the following, we examine various ways by which FRA modules can be connected to IB modules. We studied how different ranges of motion can be achieved by varying the design of the rotary joint of the assemblies. Using a state transition-based position control system, movement of the assembled modules could be controlled by regulating the pneumatic pressurization of the FRA module at the joint. These basic modules allow us to build different types of pneumatic structures. In this paper, using IB and FRA modules of various dimensions, we constructed a soft robotic limb with an end effector, which can be attached to wheelchairs to provide assistive grasping functions for users with disabilities.

## 1. Introduction

Geometry—the study of points, lines, and angles—is an extensive branch of mathematics that is deeply ingrained in our daily lives and the objects that surround us. Shape is one of the most basic ways by which we identify and characterize objects. According to the book of *Euclid’s Elements* by Euclid of Alexandria, various types of polygons exist to describe shapes of different forms, and the simplest one of them all would be trilateral polygon, an amalgamation of which can be used to derive more complex polygons [1]. It is interesting to note that, throughout civilization, triangles have widely been implemented in architecture. From the construction of bridges [2] to pyramids [3], the structures were characterized by triangular supporting elements. Given such extensive application of this specific geometry, it would be rather intriguing to explore how this particular figure could be further extended in the development of soft robots. In the field of soft robots where actuators are designed to activate into shapes of various form, it might be interesting to investigate how to develop a simple actuator unit of basic polygonal morphology, which can be used as the rudimentary cell structure to build larger assemblies of more complex shapes.

Fluidic elastomeric actuators (FEAs) of soft robots are commonly designed to be expanded or elongated in shape, with an internal pneumatic chamber [4]. Upon pressurization, the fluidic chambers inflate and FEAs achieve various forms of actuation, such as twisting, curling, and elongation [5], which intend to imitate the movement of natural beings, such as octopuses [6], snakes [7], and caterpillars [8]. It has been widely reported in literature that FEAs are designed with pneu-nets, which are inner channels that form actuation pathways for pressurized fluid, such as water and air [9]. The common way of fabricating FEAs would be via molding of siloxane-based polymers such as polydimethylsiloxane (PDMS) and silicone rubber. In order to ensure a correct form of actuation, reinforcement materials such as stiffer elastomeric polymer or woven nonstretchable fibers are included in the design as strain limiting layer [10,11]. The thickness of the fluidic chamber wall and reinforcement materials regulate the variation in stiffness of the fluidic chamber wall of FEA, which would in turn determine the actuation performance of the FEA. Lately, 3D printing has been used as a new way of fabricating soft robots, whereby the actuator is designed with 3D computer-aided design (CAD) software. The design is then fabricated using low-cost consumer-grade printers at varying fill capacity to create fluidic pathways [12,13]. A top–down fabrication approach is generally adopted for soft robots, whereby each actuator is custom-designed for target application and actuation form [14].

In addition, modular soft robots have also been developed for various applications and actuation forms [14,15,16,17]. Modularity aims to simplify the fabrication process and troubleshooting process. If damages were to occur to the module-based soft robot structure, the repair process would be simpler by replacing the faulty module. In this paper, we further explored the modular concept for developing soft pneumatic actuator modules, which exhibit shape morphology that is analogous to basic geometrical polygons.

## 2. Related Works

Modular soft robots were previously discussed by Onal and Rus, who designed and fabricated FEA modules, which can be arranged in serial, parallel, and hybrid configuration [15]. However, elastomer-based modules are fabricated by molding of liquid silicone polymer, which solidifies into the desired form upon curing. In order to attach the modules, a similar technique was applied which made the process irreversible, as the cured modules cannot be detached from one another. Furthermore, loss of mechanical energy, which would otherwise be used in force or torque generation, would occur due to deformation of elastic material. There is a limitation on the compatibility of the actuator, as the air chambers need to be designed and fabricated with minimum thickness to assure a correct form of actuation [16]. Lee et al. explored an alternative way of designing modular soft robots [14]. A comprehensive design collection of modules was presented, each of which have diverse functions—motion generation, air distribution, and connection. By selecting and arranging modules of different functions, the user is able to develop soft robots of any desired shapes or functions, such as gripper or locomotion. The actuators modules were fabricated using either multimaterial 3D-printing or molding. Each module is designed with a pneumatic chamber, and the wall thickness is varied to permit different actuation modes. Three mechanical connection mechanisms, which are screw thread, push fitting, and bi-stable junction, were presented and discussed. Each pneumatic module is fabricated with hollow connectors at either end to allow the modules to be easily attached and detached to each other by mechanical means and hence, upon connection, a central fluidic pathway is formed within the assembled modules. However, addition of extruding mechanical connectors compromises the intrinsic soft nature of the actuators and the maximum engagement pressure [17].

In this paper, a simple modular design concept is adopted, whereby triangular and rectangular shape primitives are introduced—the inflatable beam (IB) module and fabric-based rotary actuator (FRA) module. Fabric material is used to fabricate these soft pneumatic actuator modules. Fabric-based soft actuators have begun to emerge lately for various applications, such as robotic grippers or wearable rehabilitation devices [18]. The thin nature of fabric sheet allows us to create pneumatic actuators with walls which are thinner than those of silicone-based actuators, and yet capable of generating comparable performance. In contrast to silicone actuators whose fluidic chambers are fabricated using custom-shaped molds and liquid elastomeric polymers, the sheets of fabric-based actuators can be folded and sealed into pleats, which would serve as pneumatic chambers. The propagation of actuating motion is determined by the characteristics of the pleats—dimensions, location, and number of the pleats. In addition, a fabric-based fabrication protocol enables scaling up of actuator designs, without compromising on the convenience of fabrication protocol and required time [19,20,21]. Compared to 3D-printed actuators [13], fabric-based actuators require a lower range of pneumatic pressure to function. The contributions of the paper as follows: (1) A simple modular design concept using shape primitives that are inspired by basic geometrical polygons, and (2) construction of larger pneumatic structures using fabric-based modules which can be assembled and disassembled. In the following sections, we discuss the design concept of the modules, identify and characterize configurations of the modules, implement a position control system for the assembled modules, and highlight a possible application of the modules. 

## 3. Materials and Methods 

**Figure 1:**
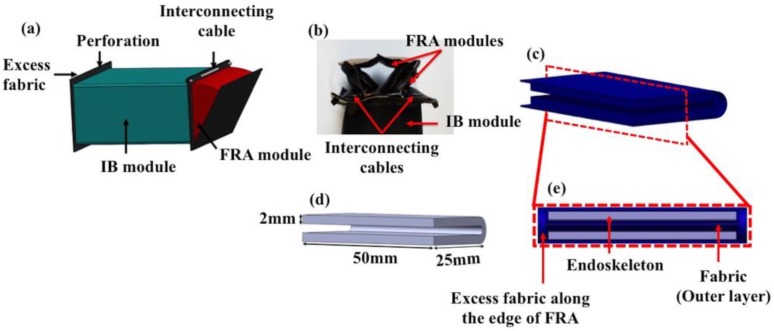
(**a**) Illustration of the fabric-based rotary actuator (FRA) and inflatable beam (IB) module assembly, (**b**) Photo of end-effector to illustrate the connection of FRA and IB modules, (**c**) FRA module in inactive state, (**d**) Ninjaflex endoskeleton, and (**e**) enlarged cross-sectional view of FRA with internal Ninjaflex endoskeleton.

Two actuator shape primitives were designed—the IB module and the FRA module (Figure 1). The IB module was designed in a rectangular block form to provide structural function in the assembly of soft robots, whereas the FRA module provides mobility function to the soft robots. When pressurized, the IB module will inflate into a rigid beam which can be used as an extension for the network of a modular-based pneumatic structure. FRAs are designed with origami-inspired V-shaped pleats which remain folded when they are deflated. Upon pneumatic pressurization, the inner pleats inflate, which causes the FRAs to open up. Due to pleated design, FRA assumes a triangular outline in both pressurized and depressurized state. The dimensions of its triangular form, however, change based on the internal pneumatic pressure. Therefore, by regulating the pneumatic pressure, the FRA form could be controlled. The opening and closing motion of FRA enable the module to be implemented in larger pneumatic structures as rotary joint modules. 

Fabric was used to fabricate the modules and it was coated with thermoplastic polyurethane (Jiaxing Inch ECO Materials Co. LTD, Zhejiang, China). By inducing heat or ultrasonic acoustic vibrations on multiple layers of stacked fabrics, the polyurethane coating on the layers will melt and be welded together. In this way, airtight pneumatic structures can be formed. However, the fabric materials are not of hyperelastic nature, as compared to the rubber elastomers that are used to develop soft robotic structures in previous works. Hence, when deactivated, the fabric-based module takes longer to return to its default state. Therefore, to solve this limitation, the FRA module was fitted with a C-shaped frame (Figure 1d), which was fabricated using Ninjaflex filament (NinjaTek, Manheim, PA, USA) via fused deposition modeling on a LulzBot TAZ 6 at 100% fill capacity. The inner C-shaped frame serves as the endoskeleton of the module, which improves the passive actuation process of the module when it is deactivated. This ensures consistent and fast return of the module to its inactive state.

**Figure 2:**
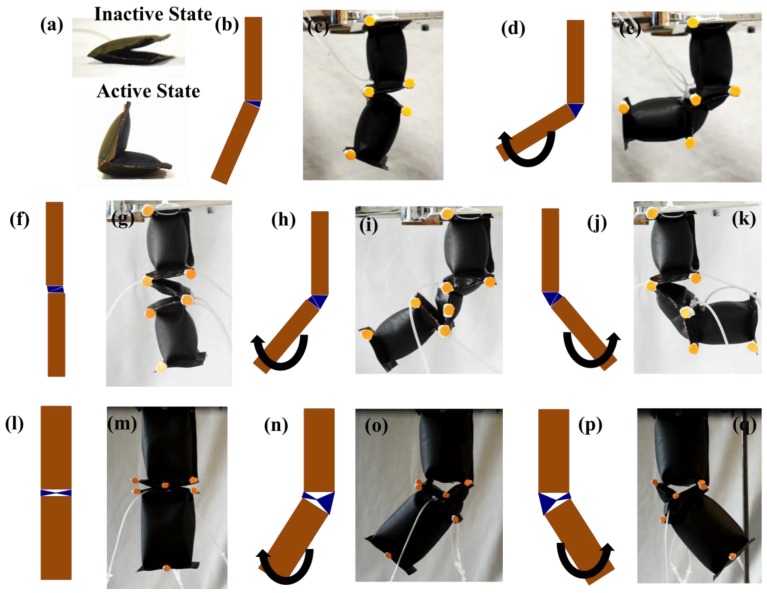
(**a**) One FRA module in depressurized (inactive) state and pressurized (active) state. (**b**–**q**) show assemblies with different configurations of FRA modules with IB modules and the active and inactive states of the assemblies. Active state refers to pneumatic pressurization of FRA module, which rotates the assembly. (**b**–**e**) One FRA module and two IB modules (SNG assembly), (**f**–**k**) two FRA modules connected in series and two IB modules (SRS assembly), (**l**–**q**) two FRA modules connected in parallel and two IB modules (PLL assembly).

FRA modules and IB modules can be assembled in various configurations. The basic assembly would consist of a single FRA module and two IB modules which would be denoted as SNG assembly in subsequent sections (Figure 2b–e). Intermediate assembly would consist of two IB modules and two FRA modules assembled in series (Figure 2f–k) or in parallel (Figure 2l–q) which will be respectively denoted as SRS assembly and PLL assembly in subsequent sections. In the following, the static response of assemblies to supplied pneumatic pressure was analyzed. Three assemblies with different configuration of FRA modules at the rotary joint (SNG assembly, SRS assembly and PLL assembly) were fabricated. The FRA modules were fabricated to the size of 50 mm by 25 mm. The attached IB modules were fabricated to be of 50 mm by 25 mm by 50 mm. The former dimensions were chosen for the convenience of the fabrication protocol. IB modules were fabricated to be of the same dimensions as FRA modules to ensure uniform distribution of force. To enable development of reconfigurable pneumatic structures, the ends of IB modules and FRA modules were fabricated with excess of fabric (5 mm), which was perforated with holes of 2 mm diameter (Figure 1a,b). This allows IB modules and FRA modules to be interlaced with each other by weaving cables through the aligned perforation of adjacent modules. This enables assembly and disassembly of IB modules and FRA modules. Unlike previously reported modular concepts, there would be no central fluidic pathways within the structure of connected modules. Each module has its own pneumatic inlet, and a pneumatic network is formed by linking the inlet tubes of different modules using commercially available connectors. Hence, it removes the need to create fluid directing units, and there could be a selective activation of modules within the pneumatic structure by selectively forming multiple pneumatic networks. 

## 4. Experiments

### 4.1. Deflection Behavior of IB Module

**Figure 3:**
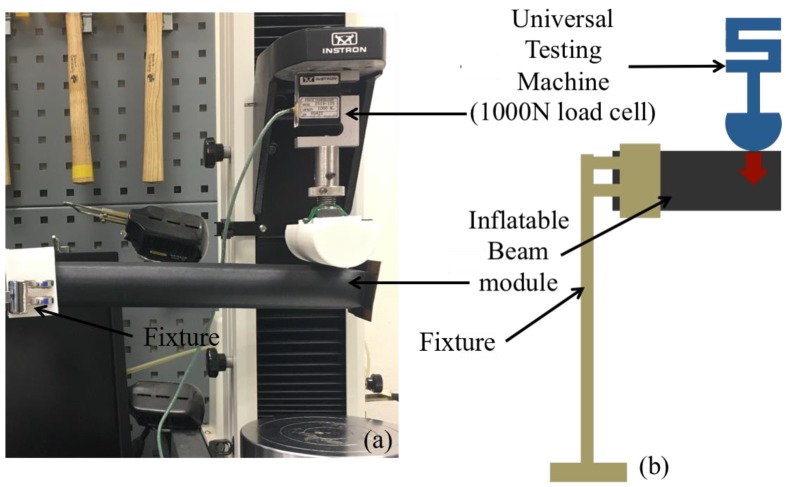
Photo (**a**) and illustration (**b**) of deflection–load test set-up for the IB module.

Wrinkling effect is a common phenomenon in textile-based pneumatic structures, which subsequently affects the stiffness of the structure and its load supporting capacity. Wrinkling occurs on the concave side of the inflated textile structure, where the textile is unable to resist compressive loads. In the wrinkled region, the bending stress due to the applied moment equilibrates axial stress in the textile layer [22]. In this paper, we focus on the load bearing capability of IB module in its initial unwrinkled phase. Most of the applications require IB module to have minimal vertical deflection when the external load is applied on it. For instance, in the subsequent sections, we illustrate a portable pneumatic limb structure that has an end-effector that enables it to grasp objects in its surrounding environment. In this case, minimal vertical deflection of the IB module is essential to allow the limb to efficiently transfer objects from one location to another. For this experiment, the IB module was fabricated to be 200 mm in length and 25 mm in diameter when inflated. One end of the beam was fixed onto a rigid platform, while the other end of the beam was placed in contact with a load cell (1000 N) of Instron Universal Tester 3345 (Instron, Norwood, MA, USA) (Figure 3). When the test begins, the load cell moves in vertical displacement (Figure 3). Deflection of the beam and the corresponding load exerted by the load cell is recorded by Instron machine. Two sets of deflection behaviors were observed, when the IB module is pressurized at 10 kPa and 20 kPa.

### 4.2. Angular Displacement Profile of Assembled Modules

The displacement characteristics of assembled modules due to pneumatic pressurization of the FRA module would vary based on the number and configuration of FRA modules at the joint. Hence, it is essential to understand the range of motion of assemblies of different configurations (SNG assembly, SRS assembly, and PLL assembly) (Figure 2). Based on this information, appropriate assemblies can be chosen and applied to form different pneumatic structures of variable functions and uses. IB modules of the assembly are supplied with constant pneumatic pressure of 20 kPa. The IB module at one end of the assembly is fixed and the second IB module at the other end is allowed to move freely upon activation of the FRA module at the joint. Changes in angle displacement of the assembly due to actuating the FRA module are recorded at different pressure levels. The FRA module at the rotary joint is pneumatically actuated from 0 kPa to 50 kPa. Pneumatic pressurization causes the FRA module to expand and deform. However, upon removal of the pneumatic source, the module returns to its original state.

### 4.3. Stiffness of Assembled Modules

**Figure 4:**
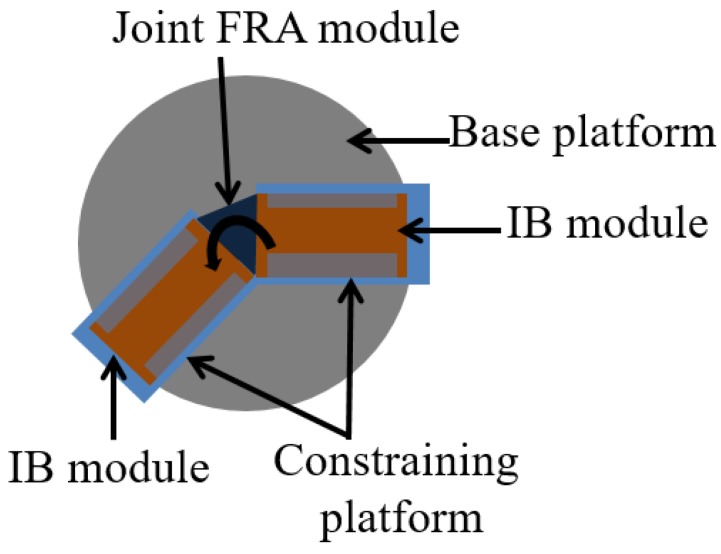
Illustration of passive resistive torque measurement set up of the SNG assembly.

Joint stiffness of assembly is introduced by the intrinsic resistance of the FRA module to rotational efforts caused by pneumatic pressurization. The joint stiffness is obtained by measuring the passive torque output of the assembled modules at different actuation angles. SNG assembly with a single FRA module is loaded onto the test platform, whereby the IB module at one end of the assembly is fixed and the second IB module at the other end is moved to specific joint angles (Figure 4), during which the joint FRA module remains depressurized. A torque sensor (Forsentek, Shenzhen, China) is used to record the passive torque output of the assembly, from which joint stiffness can be approximated as a function of joint angles.

## 5. Results and Discussion

### 5.1. Deflection Behavior of IB Module

**Figure 5:**
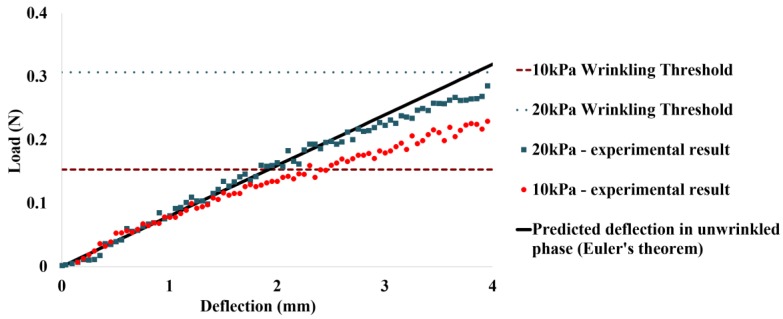
Load–deflection behavior of the IB module.

Figure 5 shows the deflection behavior of the IB module in response to applied load, when it is supplied with 10 kPa and 20 kPa of pneumatic pressure. Nylon fabric forms the external skin of the IB module and is isotropic in nature (Young’s Modulus = 34700 N/m). Upon application of load, the beam deflects, and this behavior is dependent on the elastic modulus of the fabric and the dimensions of the beam. Deflection of the beam would undergo three phases [22]. In the initial phase of beam deflection, the external fabric layer of the beam is unwrinkled. The beam would exhibit a linear relationship between deflection of the beam and applied load. This relationship can be described using conventional solid elastic beam and Euler beam theory [22]. In the subsequent phase, wrinkling of the external skin (nylon fabric) of the beam would occur, when the applied load reaches the wrinkling point [22]. Increase in magnitude of applied load leads to propagation of wrinkling of the nylon fabric until it reaches the collapse point, whereby the beam is no longer able to support the applied load. This experiment measures the load bearing capacity of the inflatable beam before wrinkling occurs. Based on Euler theory [22], deflection of the beam in the unwrinkled phase can be modeled as a regression line with a gradient of 0.0799 N/mm, which is also indicated in Figure 5.

The load bearing capacity of the IB module is independent of inflation pressure during the initial unwrinkled phase. Hence, the slope of the regression line, which relates deflection of the IB module with respect to applied load, remains the same for different pneumatic pressures [23]. However, the threshold of load, which is required for wrinkling to occur, is affected by input pneumatic pressure. The wrinkling load threshold of beam increases, when the supplied pneumatic pressure to the beam increases from 10 kPa to 20 kPa. For an inflated beam of 25 mm in diameter and 200 mm in length, it is able to theoretically withstand a wrinkling load of 0.307 N, when it is supplied with 20 kPa of pressurized air. When input pneumatic pressure is reduced to 10 kPa, the wrinkling load threshold reduces to 0.153 N (Figure 5). At this point, the experimental data also begin to deviate from the expected linear regression model for load bearing capacity in the unwrinkled phase. 

### 5.2. Angular Displacement Profile of Assembled Modules

**Figure 6:**
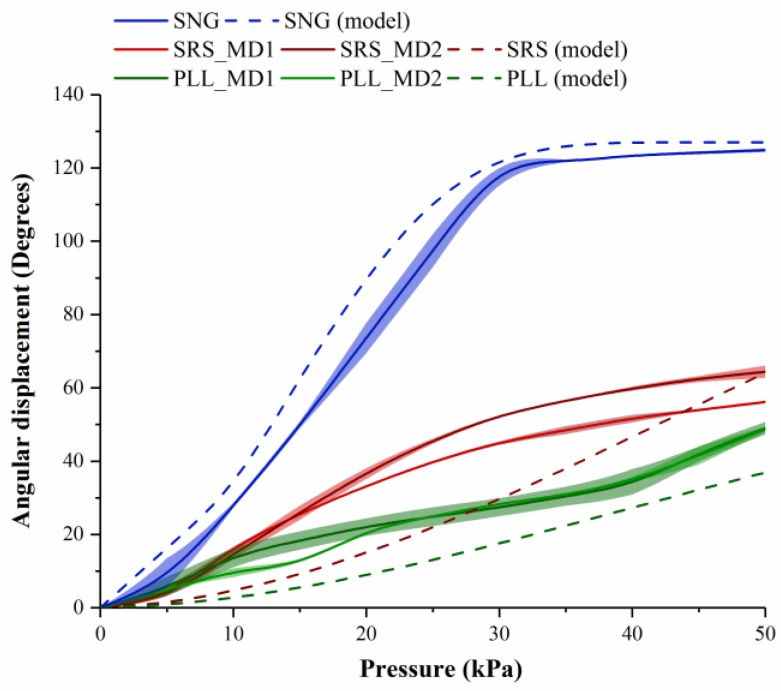
Experimental and predicted angular displacement profiles of assemblies—SNG assembly, SRS assembly, and PLL assembly. SRS and PLL assemblies have two FRA modules, each of which is pressurized selectively. Hence, two sets of angular displacement profiles are presented for the SRS (SRS_MD1 and SRS_MD2) and PLL assembly (PLL_MD1 and PLL_MD2). MD1 refers to the state whereby the first FRA module is pressurized and the second FRA module depressurized. MD2 refers to the state whereby the first FRA module is depressurized and the second FRA module pressurized.

Angular displacement of assembled modules is generated by the unfolding action of the joint FRA module. SNG assembly exhibits higher angular displacement compared to assemblies with double FRA modules (SRS assembly and PLL assembly). For the SNG assembly, maximum angular displacement (124.86 ± 0.50°) is reached at 30 kPa, beyond which a plateau is observed upon further increment in pneumatic pressure (Figure 6). This is because the unfolding of the FRA module is limited by the length of inner folded fabric layer. The SRS assembly exhibits maximum angular displacement of 56.14 ± 0.26° and 64.39 ± 1.66° in either direction. The PLL assembly exhibits maximum angular displacement of 48.98 ± 1.71° and 48.67 ± 0.74° in either direction.

**Figure 7:**
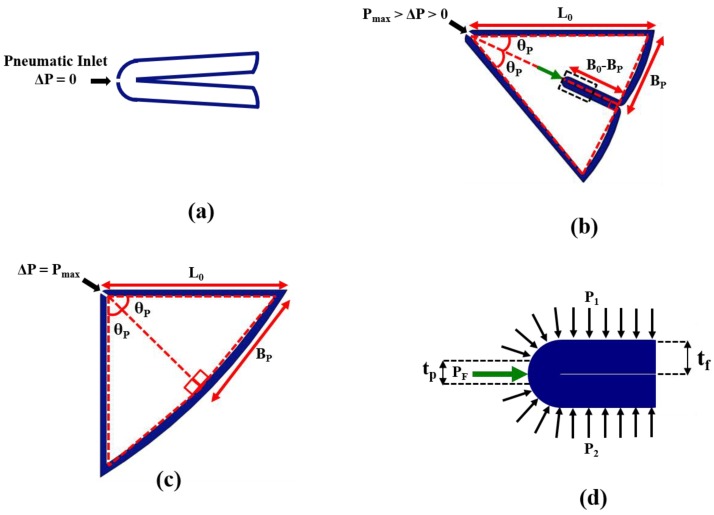
(**a**–**c**) Simplified illustration of FRA module outline at different pneumatic pressure, (**d**) enclosed view of the dotted section in (**b**).

The morphology of the actuated FRA module is assumed to adopt a simplified outline with resemblance of a triangle. Upon pressurization, the unfolded section of the inner fabric layer forms a triangular outline with the outer fabric layer. As the FRA module expands upon pressurization, the outline of the module also increases with pneumatic pressure. The unfolding action of the activated FRA module causes a respective change in the angulation. It is assumed that the external fabric layer is inextensible and that during the initial unpressurized state, the external and internal layer form rectilinear edges. Figure 7 shows a simplified illustration of changes in the FRA outline during pressurization. When pressurization begins and the inner pleats of FRA begin to unfold, the external and internal layers form an angle with respect to each other. Compression force, which is generated along the inner fabric layer of FRA due to pressurized air, is translated into a pushing force due to the way the inner layer is folded. Increasing pneumatic pressure within the FRA results in corresponding increment in the pushing force. This causes FRA to unfold further, until force equilibrium is reached along the inner fabric layer of the module. The inner fold of the FRA module is approximated as a wide beam [24], and the corresponding change in unfolded length of inner fabric layer at respective pneumatic pressure is expressed by the following relation:

∆P = P_1_ = P_2_ = P_F_, (1)

B_P_ = ((P_F_·B_0_(B_0_ − B_P-1_))/α·t_p_·E) + B_P-1_. (2)

**Figure 8:**
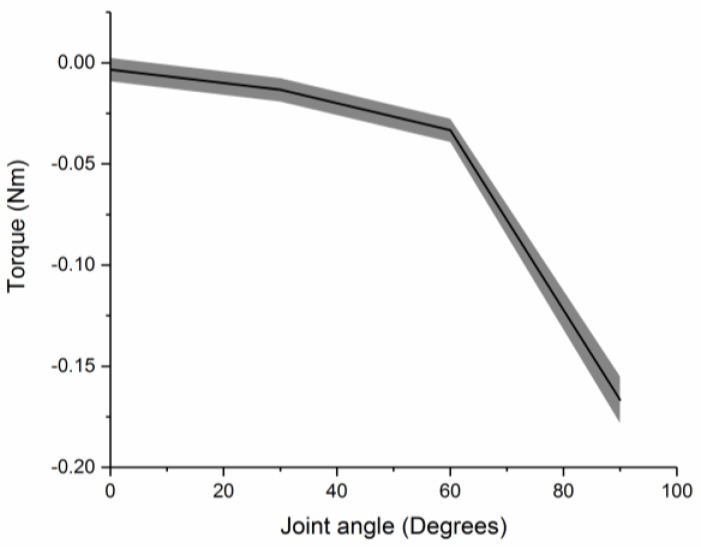
Measurement of resistive torque generated by SNG assembly (one FRA module and two IB modules).

ΔP is pneumatic pressure supplied to the FRA module. P_1_ and P_2_ refer to pressure along the inner surface of FRA during the equilibrium state. P_F_ is pressure applied along the inner folded edge to generate pushing force, which causes the FRA module to unfold. B_0_ is the breadth of the inner fabric layer of the FRA module. B_P_ is the length of the inner layer, which is unfolded at current pneumatic pressure (ΔP), whereas B_P-1_ is the length of the inner layer, which has previously been unfolded at lower pneumatic pressure. E refers to Young’s Modulus of fabric [25]. α refers to the stiffness coefficient of the joint of the assembled modules. The joint stiffness coefficient is derived directly from the experiment in Section 4.3, whereby resistive torque output of the SNG assembly with one FRA module at the joint is measured at different joint angles (Figure 8). 

t_p_ is the length of the inner layer, upon which pushing force is concentrated to cause unfolding of FRA. t_p_ is assumed to be of minimum length of 0.4 mm, which is the thickness of the fabric. The range of motion of the FRA module is limited by the amount of fabric within the pleat and the inherent resistance of the Ninjaflex endoskeleton. The inner folding of fabric and Ninjaflex endoskeleton of the FRA module provides resistance to the unfolding motion of the module. This enables the module to resume its initial angle at an unactuated state. In an assembled modular structure, resistance to deformation may vary based on the number of FRA modules attached at the rotary joint. As seen in Figure 6, the angular displacements of the SRS and PLL assemblies are smaller than that of the SNG assembly. To explain this occurrence, a spring model is adopted based on the assumption that stiffness of the assemblies varies with the number and orientation of the FRA modules. Due to variation in stiffness of rotary joints, module assemblies with different configurations of FRA modules would have different ranges of motion. Rotary joints that are designed with multiple FRA modules would exhibit higher resistance. In the following, the multiple modular joint design is modeled using a parallel spring model to reflect the increase in stiffness of the joint. Due to the increase in joint resistance to rotation, the length of unfolded fabric would also decrease and, hence, the range of angular displacement.

θ_P_ = 2 sin^−1^ (B_P_/L_0_), (3)

F = κ_eq_·X, (4)

κ_eq_ = κ_1_ + κ_2_, (5)

α = κ_1_. (6)

L_0_ is the length of the inner fabric layer of the FRA module. θ_P_ refers to angular displacement of the FRA module. κ_eq_ is the equilibrium stiffness of the rotary joint of the assembled modules, whereby κ_1_ and κ_2_ refer to the stiffness of the FRA modules in the rotary joint. In intermediate rotary joint assemblies, two identical FRA modules were fabricated, whereby κ_1_ = κ_2_. 

Figure 6 also shows the expected angular displacement profile of module assemblies based on the mathematical assumption and model (SNG (model), SRS (model), and PLL (model)). For assembly with one FRA joint module (SNG assembly), a similar angular displacement profile was observed between the experimental and model-derived data. However, discrepancy was observed between the expected and measured displacement profiles of assemblies with intermediate joint assemblies (SRS and PLL assemblies). This discrepancy is due to the simplified assumptions of the spring model. Additional parameters such as resistance to rotation due to stacking of multiple layers of fabric may need to be considered. In order to more accurately predict the motion of intermediate assemblies, a more complex spring model would be required. In general, the displacement profiles of all three assemblies (SNG, SRS, and PLL assemblies) are comparable between the experimental results and model. The SNG assembly showed a larger displacement, followed by an SRS assembly and then PLL assembly. 

## 6. Design of Control System

**Figure 9:**
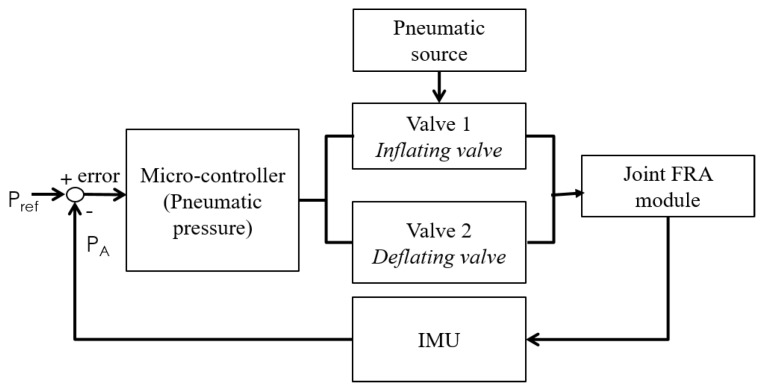
Position control of the module assembly.

A state machine was developed as a position control system for the assembled modules, whereby pneumatic pressure within the FRA modules at the rotary joint is regulated to control the position of the linked IB modules. Zhao et al. developed state machine controllers for the elastomeric fingers in the soft orthotic glove [26], whereby optical fiber sensors were used to provide feedback of the curvature of the elastomeric actuators. Similarly, in this paper, a state machine controller was designed for each FRA module in the joint of the assembled modules. For each state machine controller, an integrated valve system was designed with two miniature normally-closed solenoid valves (X-Valve, Parker Hannifin Corp., OH, USA), which have a nominal response time of 20 ms (Figure 9). One valve was used as the inflating valve, which links the actuator to the pneumatic source, and the other valve was used as the deflating valve, which links the actuator to the atmosphere. The integrated valve system enables (1) air flow in to the actuator and (2) air flow out of the actuator and (3) contains air within the actuator.

**Figure 10:**
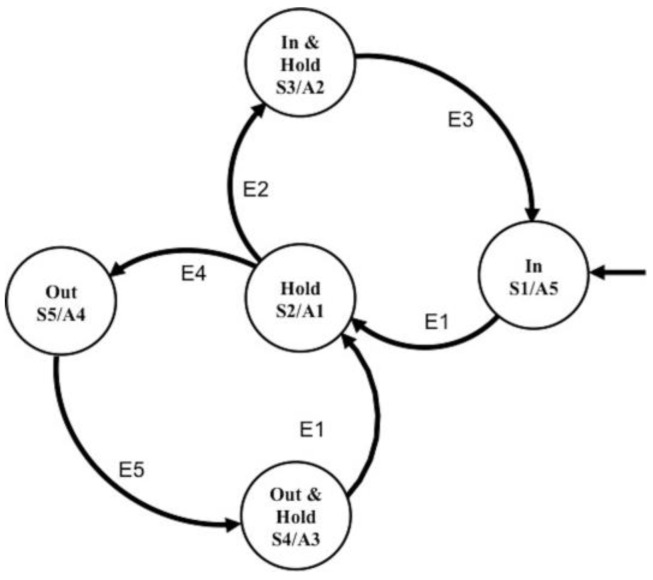
State transition map for the position control algorithm.

The state machine controller was designed with five control states, each of which requires different actions of the two valves in the integrated valve system (Figure 10). Transition between states is determined by the percentage error of the measured angle with respect to the set angle (ε) and the location of ε in the preset error margin (K_a_, K_b_, and K_c_). K_a_ determines the acceptable margin of error for ε. K_b_ and K_c_ determine the allowable threshold of the error margin. In the following test, the control parameters were selected to be as follows: K_a_ = 0.05, K_b_ = 0.01, and K_c_ = −0.01. When the error thresholds are exceeded, the controller would switch to either an “in” or “out” state, and inflation and deflation of the actuator occur accordingly. At each control state, the opening and closing duration of respective valves are controlled using pulse width modulation, the duty cycle of which is varied based on the preset time variables (X and Y).

An inertial measurement unit (IMU) (Pololu Corp., Las Vegas, NV, USA) was used to provide sensor feedback for the measured angle of the IB module that is linked to the actuating FRA module. The IMU is placed at the distal end of the freely moving IB module along the central axis. By tracking the orientation of IMU, the angle of the actuating IB module can be tracked. Due to the actuation form of FRA, the movement of the attached IB module would be assumed to be a single plane and that the movement of the assembled modules would be minimal in other planes. Hence, based on the orientation of IMU on the actuator, the estimated roll angle of IMU output data is used to provide joint angular displacement feedback to the state machine.

**Figure 11:**
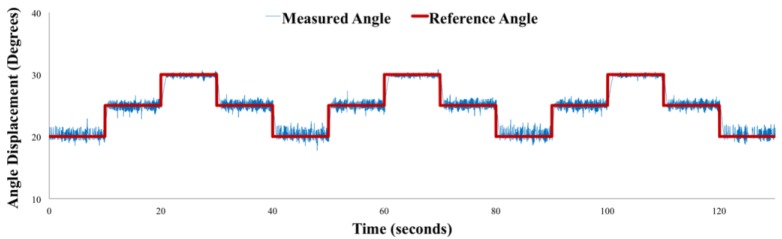
Position control step response.

Assembly with FRA modules in parallel configuration (PLL assembly) was chosen to analyze the step response of the position controller (Figure 11). For the step position tracking experiment, the reference angle was set from 20° to 30°, with increments of 5°. For the step response, the rise time was 550 ms and the settling time was 660 ms. The steady state error was 1.30%. Despite the “hold” control state, the assembly is observed to oscillate around the target value. 

Amidst steady state oscillation, a higher magnitude of oscillation is observed at lower reference angles (20°and 25°) than at 30°. This is because the pneumatic source is supplying air at 35 kPa. Hence, when the inflating valve opens, the inflow of air within the actuator is at 35 kPa, which would cause the actuator to displace at a higher angle than the expected value. An inner pressure control loop would have to be included to automatically adjust the supplied pressure of the pneumatic source.

## 7. Implementation

**Figure 12:**
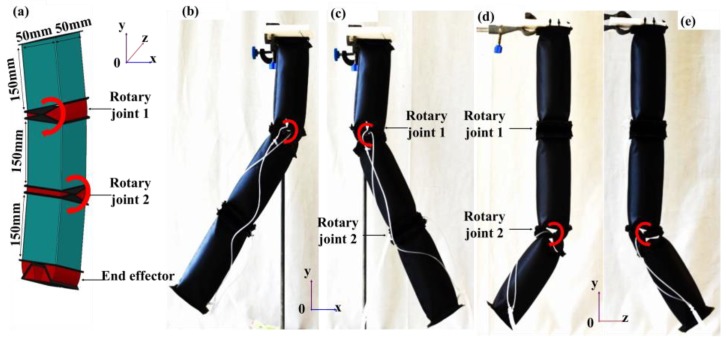
(**a**) Illustration of textile-based robotic arm (TERA). (**b**–**e**) The limb segment of TERA.

Based on the characterization tests, a trade-off exists regarding the range of motion and joint stiffness for the different module assemblies. Hence, to construct larger pneumatic structures, the most suitable form of assembly would have to be chosen based on the requirement of the end structure. The following demonstrates a possible application of pneumatic actuator modules in the construction of soft robotic limb—also known as textile-based robotic arm (TERA). Previously, Otherlab developed a soft robotic limb, which was designed with bellow shaped joint actuators. The multiple folding of the bellow shaped actuator makes the actuator prone to wrinkling effect, which can reduce the stiffness of the actuator and its ability to support load [27]. TERA is constructed with FRA modules and IB modules, which are arranged in variable configurations and orientations along the structure (Figure 12). PLL assemblies are used to create limbs of TERA, which are able to perform bidirectional rotation. It is designed with two rotary joints, each of which has a pair of FRA modules in parallel configuration. The rotary joints of TERA are interconnected using IB modules (Figure 12). FRA modules of rotary joints are fabricated to 50% of the dimensions (50 mm by 25 mm) of linkage IB modules (50 mm by 50 mm by 150 mm).

**Figure 13:**
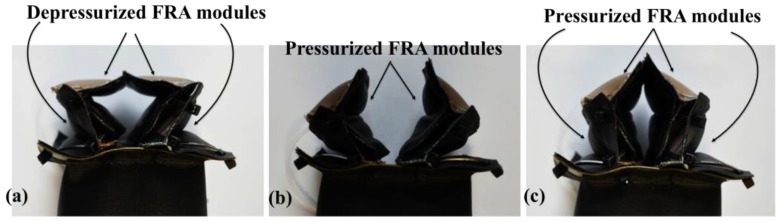
Photo of the end effector in (**a**) initial state, (**b**) intermediate state to prepare for the grasping of objects, (**c**) final state of grasping mode.

**Figure 14:**
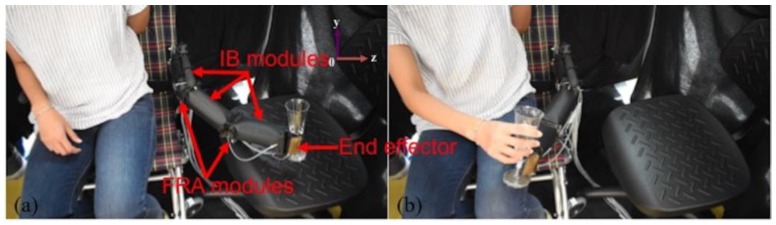
(**a,b**) Photo of TERA on a wheelchair for the disabled. Grasping and moving of a glass. The pneumatic pressure for IB modules is 20 kPa and the actuating FRA module of the respective joint is 50 kPa.

Hence, when paired in parallel configuration, the FRA modules would be fitted evenly to the adjacent IB modules. This is to ensure uniform transmission of force by the actuating FRA module in the rotary joint to the linked IB module, therefore maximizing the range of motion of the structure. The two pairs of FRA modules in the rotary joints are arranged in different orientations. Activation of FRA modules at different joints would enable movement of the robot along different orientation planes, therefore increasing the degree of freedom. Reversible linkage is formed between FRA modules and IB modules using cables. Each module is fabricated with excess fabric perimeter at either end of the module, which is perforated to create paths for weaving of interconnecting cables between adjacent modules. Should the design require alteration, the interlacing cables can be removed, and the affected modules can be rearranged to the desired conformation. As opposed to the usage of parallel configuration, the end effector of TERA is designed with two sets of FRA modules in series configuration (Figure 13), whereby FRA modules are selectively activated to initiate grasping mode. As a portable, mobile system, TERA can be used as a supernumerary limb or it can also be attached to items such as wheelchairs for the disabled and be used to perform simple grasping tasks for household items, such as glassware and stationery (Figure 14). In the future, electromyography control can be incorporated to detect the intention of the disabled user.

Compared to existing similar rigid robotic arms, a soft robotic arm can be made at lower cost and greater ease of fabrication. As it is mainly made with nylon fabric and actuated with air, the soft robotic arm is also much lower in weight. However, the payload of the soft robotic arm is smaller than that of a rigid robotic arm. Nevertheless, due to its lightweight and intrinsic soft nature, the soft robotic limb can be integrated onto structures such as wheelchairs and be deployed in close proximity with human users.

## 8. Conclusions

To give a new perspective to the conventional design and way of fabrication, we opted to design soft pneumatic modules with morphological resemblance of basic polygons of three-sided and four-sided figures—triangle and rectangle. A simplistic modular approach was adopted whereby two shape primitives were introduced—the IB module and the FRA module, which are fabricated with thermoplastic polyurethane-coated fabric. The rudimentary polygonal outline of modules enhances their versatility, for it permits construction of larger pneumatic structures of more complex shapes and actuation modes through selective congregation of multiple modules. The contributions of the paper are as follows: (1) A simple modular design concept using shape primitives that are inspired by basic geometrical polygons, and (2) construction of larger pneumatic structures using fabric-based modules, which can be assembled and disassembled. Module-based construction of pneumatic structure requires lower volume of operating fluid to function and is able to operate within a lower range of pneumatic pressure, compared to continuum actuators. Reconfigurable fabrication allows the usage of the same designs of actuators to construct more than one type of pneumatic structures. The versatility of the modules was illustrated by the construction of a soft textile-based robotic arm (TERA) with two rotary joints and an end-effector, all of which were developed using the two essential modules. TERA is a lightweight, mobile system which can be attached to objects such as wheelchairs for the disabled to assist with grasping tasks.
